# Role of EZH2 in adipogenesis and obesity: Current state of the art and implications – A review

**DOI:** 10.1097/MD.0000000000030344

**Published:** 2022-09-09

**Authors:** Haixia Wang

**Affiliations:** a Zhejiang Changzheng Vocational and Technical College, Hangzhou, P. R. China.

**Keywords:** adipogenesis, epigenetics, EZH2, obesity

## Abstract

Obesity is characterized by excessive accumulation of adiposity and has been implicated in a strong predisposition to metabolic disorders and cancer, constituting one of the major public health issues worldwide. The formation of new mature adipocytes through differentiation of progenitor or precursor cells during adipogenesis can lead to the expansion of adipose tissue. Recent studies have revealed that the intrinsic risk of obesity arises not only through genetic variants but also through epigenetic predisposition. Enhancer of zeste homolog 2 (EZH2) is an enzymatic catalytic component of polycomb repressive complex 2 that acts as an epigenetic modulator in the regulation of gene expression. EZH2 can modulate the expression of its target genes by the trimethylation of Lys-27 in histone 3 or methylation of non-histone proteins. Emerging evidence has shown the important role played by EZH2 in adipogenesis and obesity. This review provides the latest knowledge about the involvement of EZH2 in the process of adipogenesis and obesity involving adipocyte differentiation, extract key concepts, and highlight open questions toward a better understanding of EZH2 function and the molecular mechanisms underlying obesity.

## 1. Introduction

Obesity is a chronic disease caused by excessive or abnormal accumulation of white adipose tissue, resulting from the malfunction of the energy storage machinery. The increasing prevalence of overweight and obesity has become a pressing public health challenge, placing a severe global health burden.^[1]^ According to the World Health Organization, approximately 20% of adults worldwide are diagnosed with obesity by 2025.^[2,3]^ Overweight and obesity are well-documented risk factors for chronic diseases, including type II diabetes, heart disease, stroke, hypertension, and certain cancers. Reaching and maintaining a healthy weight are considered risk modifiers of glucose metabolism, blood pressure, and cardiovascular function.^[4]^ The global prevalence of obesity has sparked great interest in exploring the molecular and cellular mechanisms underlying the development of obesity and discovering optimal strategies for minimizing the adverse metabolic consequences of obesity while enhancing efficient energy storage. Obesity is characterized by deregulated adipogenesis and adipocyte differentiation, which are complex processes involving the conversion of undifferentiated mesenchymal stromal cells and pre-adipocytes into mature adipocytes with changes in morphology, gene expression profile, regulatory peptide synthesis, and susceptibility to hormonal influences.^[3,5]^ Despite the well-established role of adipocytes in energy storage and obesity, the molecular mechanisms underlying adipogenesis and adipocyte differentiation in obesity are yet to be fully elucidated.

In recent years, epigenetic studies have extended our understanding of the steep rise in obesity rates, and a link between disturbances in epigenetic modifications and obesity has been identified. All identified genetic factors have been proven to account for only a small proportion of phenotypic changes in obesity.^[6]^ Epigenetic regulation is characterized by stable changes occurring through biochemical modifications in the DNA bases and structure of the chromosome, but not in the DNA sequence.^[7,8]^ Enhancer of zeste homolog 2 (EZH2) is a key epigenetic modulator in the family of polycomb group genes (PcGs) that includes critical epigenetic regulators that repress gene transcription. Being an enzymatic catalytic subunit of polycomb repressive complex 2 (PRC2), a component of core PcG complexes that mediate gene silencing, EZH2 primarily regulates gene expression via the trimethylation of Lys-27 in histone 3 (H3K27me3).^[9]^ EZH2 can also methylate non-histone factors to regulate downstream gene expression in a PRC2-independent manner. Accumulating evidence has shown the essential role played by EZH2 in various biological processes, including cell proliferation, apoptosis, necrosis, DNA repair, cell lineage determination, and cellular senescence. EZH2 has been implicated in multiple diseases such as cancer, obesity, and skin disease.^[10]^

This review summarizes recent advances in EZH2-mediated regulation of adipogenesis and obesity, breaking new grounds for a better and deeper understanding of the molecular mechanisms underlying adipogenesis and the occurrence and progression of obesity.

## 2. Obesity and Adipogenesis: Impact, Causes, and Signaling Pathways

The global prevalence of overweight and obesity has significantly increased over the last 3 decades, posing a great challenge for the prevention and management of chronic diseases and public health of major proportion.^[3]^ It has been shown that over 600 million adults, 340 million children, and adolescents of pediatric age are clinically affected by obesity worldwide. The deleterious effects of obesity continue into adulthood in 70% of adolescents. Obesity is a relapsing chronic disease characterized by increased pathological accumulation of adipose tissue caused by dysregulated energy homeostasis pathways and is one of the major driving forces in chronic diseases such as hypertension, type II diabetes, metabolic syndrome, coronary heart disease, stroke, and cancer.^[1,11]^ The increase in adipose tissue mass can be caused by the enlargement of adipocyte size or by the expansion of adipocyte numbers resulting from the process of cell differentiation into adipocytes.^[1]^ Despite extensive research exploring obesity, it remains a chronic multifactorial disease with a complex etiology. Genetic, epigenetic, environmental, and socioeconomic factors are involved in the adipogenic process of obesity.^[3]^ Due to the limitations on the effectiveness of conventional therapeutic regimens for obesity, including pharmacological treatment and lifestyle interventions, the development of novel approaches for treating obesity based on updated discoveries on the molecular mechanisms underlying obesity remains profoundly necessary.^[12]^

Adipogenesis is a biological process that enables adipose tissue expansion, whereby mesenchymal stem cells (MSCs) or fibroblast-like progenitor cells differentiate into mature triglyceride-filled adipocytes.^[13]^ Adipose tissue, previously defined as the tissue exhibiting the ability to package triglycerides into large lipid droplets for energy storage, is currently considered one of the largest metabolic and endocrine organs in the regulation of whole-body energy homeostasis, as well as a highly dynamic tissue for cellular and metabolic reactions. Conventionally, adipocytes react to systemic signals for energy storage and release. During glucose limitation, adipocytes are triggered by glucagon or noradrenaline to release fatty acids into the circulation, which are utilized for fuel by organs such as the liver and skeletal muscle. Under overnutrition conditions, adipocytes are adapted to store excess nutrients in the form of lipid droplets in response to the regulation of elevated blood glucose by insulin.^[14]^

Pathological adipose tissue expansion during the progression of obesity is often accompanied by adipose tissue dysfunction, such as dysregulated inflammation, excessive secretion of adipocyte-derived factors, disrupted lipid metabolism, and pathologic neovascularization, resulting in prominent influences on the maintenance of body homeostasis. In obesity-induced metabolic alterations, adipocytes lose the ability to react properly to regulatory signals, most strikingly insulin, leading to increases in the levels of blood glucose and lipids and a deregulated transcriptional profile with an enhanced production of collagen and an increase in necrosis, which consequently promotes the levels of pro-inflammatory cytokines.^[14,15]^ In the process of forming new adipocytes, MSCs that exhibit the potential to differentiate into various cell lineages, including adipocytes, osteoblasts, and myocytes, can restrict their fate commitment toward the adipocyte lineage. The capacity for adipocyte differentiation in adipose tissue cellularity remains in adult humans, with a significant proportion of multipotent cells capable of undergoing adipocyte differentiation.^[16,17]^

**Figure 1.**
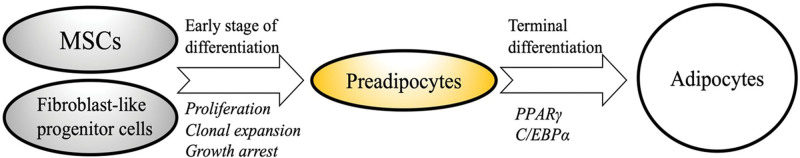
The schematic view of formation of new mature adipocytes from MSCs or progenitor cells. MSCs = mesenchymal stem cells.

During the early stage of adipocyte differentiation, preadipocytes originate from MSCs or progenitor cells that restrict their fate to the adipocyte lineage through cell proliferation, clonal expansion, and growth arrest, without significant changes in phenotype.^[17]^ Early adipocyte commitment factors that drive the differentiation process include bone morphogenetic protein 2 (BMP2), BMP4, BMP7, and the binding proteins CCAAT/enhancer β (C/EBPβ) and C/EBPδ. The upregulation and activation of these factors subsequently initiate terminal differentiation by activating the transcription of peroxisome proliferator-activated receptor-γ (PPARγ) and C/EBPα, which play central roles in the late phase of adipogenesis. In the subsequent differentiation stage, committed preadipocytes undergo terminal differentiation into adipocytes, accompanied by remarkable changes in morphology and phenotype, upregulation of adipocyte markers, and development of the lineage-specific capability to synthesize and secrete adipokines and lipokines.^[3,17]^ During the terminal differentiation stage, the activation of PPARγ by binding to its ligands induces morphological changes and the formation of a gene expression profile specific to mature adipocytes. There are various endogenous ligands for PPARγ, including polyunsaturated fatty acids, eicosanoids, and prostaglandins. One of the most critical events following PPARγ activation is the process of tuning the expression of C/EBPα, which is a potent factor driving adipocyte differentiation. PPARγ and C/EBPα then form a positive feedback loop by activating the transcription of each other via direct binding to the promoter region, cooperating synergistically in full activation of mature adipocyte program, including the expression of insulin receptor, adipocyte protein 2, adiponectin, Krox20, Kruppel-like factors, and proteins involved in lipolysis and lipogenesis.^[3,12,18]^ A schematic view of the formation of new mature adipocytes from MSCs or progenitor cells is shown in Figure 1.

Multiple signaling pathways have been reported to be involved in adipogenesis, such as insulin, glucocorticoid, BMP, Wnt, and Hedgehog signaling. In the insulin signaling pathway, which enhances glucose uptake in tissues for either usage or storage, insulin triggers signaling by binding to the insulin receptor or insulin-like growth factor 1 receptor expressed on cells in adipose tissue. In the subsequent intracellular signal transduction, insulin receptor substrates, including insulin receptor substrate 1, PI3K, Akt 1, and Akt 2 kinase, are consequently activated. The signalling cascade then activates mTOR and forkhead proteins, driving adipocyte differentiation. Disruption of insulin signaling pathways, such as insulin deficiency, insulin receptor dysfunction, and IGF-1 receptor, results in the impairment of adipocyte differentiation.^[19,20]^ In addition to insulin signaling, the signaling cascade triggered by proadipogenic glucocorticoids also plays a substantial role in adipogenesis. During adipocyte differentiation, glucocorticoids can expedite growth arrest in the early phase, increase the insulin sensitivity of cells, and upregulate the expression of C/EBPs.^[12]^ BMPs, early adipocyte commitment factors, are known to be required for adipocyte differentiation. The binding of BMPs to BMP receptors induces an intracellular signaling cascade that leads to the phosphorylation of SMAD proteins, an upstream activator of PPARγ, thereby turning on the transcription of PPARγ.^[3,21]^ Unlike insulin, glucocorticoids, and BMPs, Wnt plays a suppressive role in adipogenesis. In preadipocytes, stabilization of β-catenin induced by Wnt signaling gives rise to the suppresses the expression of PPARγ and C/EBPα and induces a phenotypic shift toward other cell lineages such as osteoblasts and immune cells. Moreover, in the adipose tissue microenvironment, Wnt levels can be elevated by hypoxia and endothelial cells, which further limits adipogenesis at local sites.^[3,22,23]^ Hedgehog signaling is another inhibitory pathway in adipogenesis.^[22]^ Hedgehog signaling inhibits adipogenesis by inhibiting the insulin and BMP pathways. Similar to Wnt signaling, Hedgehog can induce the redirection of the fate of preadipocytes toward the osteogenic lineage.^[22,24]^ Hedgehog signaling can also repress adipogenesis and improve wound healing in response to muscle injury.^[24]^

## 3. EZH2: Biology and Functions

Epigenetic mechanisms control gene expression by adapting to the DNA regions and proteins associated with the locus. Epigenetic regulation is commonly mediated by regulatory proteins responsible for the alteration of chromatin structure, thus regulating the expression of specific genes.^[25]^ PcG proteins form a major family of epigenetic transcriptional regulators that mediate the silencing of target genes. PcG proteins can play an inhibitory role in the activation of gene transcription through the hypermethylation of histones H3 and H2A in chromatin. PRC2 is the core functional multiprotein complex formed by PcG proteins in transcriptional repression and plays an essential role in various biological processes, such as cell proliferation, apoptosis, differentiation, chromosome activity, stem cell plasticity, and cellular identification.^[25]^ The methyltransferase activity of PRC2 enables the addition of methyl groups to histone H3, thereby mediating epigenetic alterations in controlling gene expression. Constitutional inhibition, mutations, or deregulated expression of PRC2 leading to its dysfunction have been implicated in a diverse range of human diseases.^[26]^

**Figure 2.**
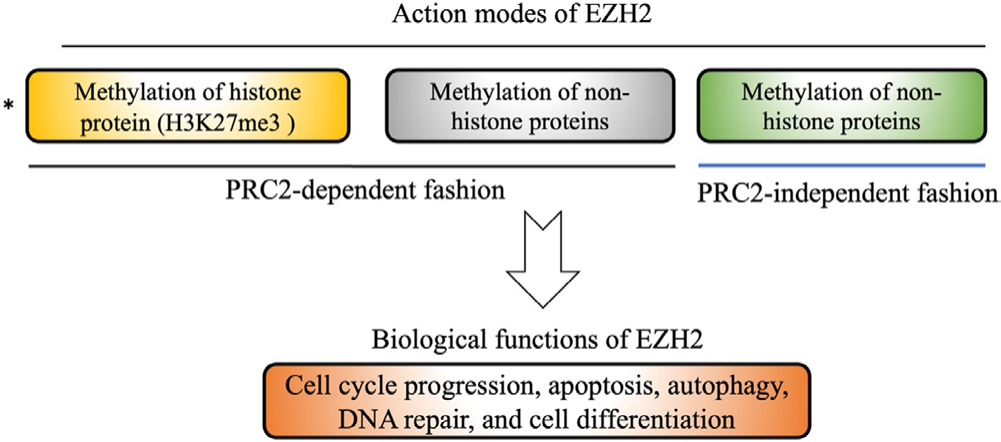
Mechanisms of actions and biological functions of EZH2. EZH2 = enhancer of zeste homolog 2.

EZH2 belongs to the PcG protein family and is a major enzymatic subunit of the PRC2 complex with 2 additional catalytic subunits, embryonic ectoderm development and suppressor of zeste 12.^[26,27]^ The gene encoding EZH2 includes 20 exons and is located on chromosome 7q35. EZH2 protein contains an embryonic ectoderm development -interaction domain, Domain I, Domain II, cysteine-rich domain, C-terminal suppressor of variegation 39, enhancer of zeste, and trithorax domain. The enhancer of zeste, and trithorax domain is responsible for EZH2 methylation activity, and the cysteine-rich domain provides a protein interaction site for assembling other subunits in the PRC2 complex.^[10]^ EZH2 acts as an S-adenosyl-L-methionine-dependent histone methyltransferase primarily via trimethylation of the lysine 27 residue at histone H3 and generates the repressive H3K27me3.^[26]^ H3K27me3 is considered a pivotal event in the epigenetic regulation of gene expression by repressing transcription activation. For example, EZH2 can bind to the promoter region of p21, a tumor suppressor gene, and mediate modifications in the form of H3K27me3, leading to silencing of p21 transcription.^[28]^ In addition to PRC2-dependent histone H3K27 methylation, EZH2 can also methylate non-histone proteins in a PRC2-dependent manner. For instance, EZH2 represses the transcription of the cardiac transcription factor GATA4 by methylating Lys 299 to inhibit p-300-mediated GATA4 acetylation.^[10]^ In addition to the PRC2-dependent pathway, PRC2-independent gene transactivation is another mode of action of EZH2. EZH2 directly methylates non-histone proteins such as STAT3 in the STAT3 signaling pathway and androgen receptors to activate downstream genes.^[10,29]^ Via the above action modes, EZH2 acts as a master regulator in various biological processes, including cell cycle progression, apoptosis, autophagy, DNA repair, cell differentiation, and so forth^[10,30,31]^ (Fig. 2).

Extensive studies have revealed the important roles of EZH2 in the onset and development of cancer and cell lineage determination during development.^[10,31]^ EZH2 has been found to be closely associated with cancer initiation, progression, metastasis, immune escape, and resistance to treatment.^[10,31,32]^ Being one of the key regulators of the cell cycle, EZH2 mutations and deregulated EZH2 expression lead to acceleration of cell proliferation and retardation of apoptosis, which are related to carcinogenesis. EZH2 enhances cancer metastasis by regulating epithelial-mesenchymal transition and tumor angiogenesis via paracrine circuits. EZH2 also contributes to drug resistance in various types of cancer through transcriptional regulation. Aberrant upregulation and gain-of-function mutations in EZH2 in cancer play a suppressive role in regulating the expression of tumor suppressors.^[33,34]^ EZH2 overexpression is positively correlated with tumor growth, advanced stage, lymphatic invasion, poor prognosis, and overall survival in cancer patients.^[35–38]^ Increasing evidence has shown the critical role played by EZH2 in cell fate decision with epigenetic mechanisms involved, in which the behaviors of the cells to grow, proliferate, differentiate, and develop morphological profiles are closely integrated.^[31]^ Given that epigenetic modifications can be inherited and are important for the maintenance of cell identity, cell fate decisions from progenitor cells to differentiated specialized types of cells are profoundly influenced by epigenetic regulation. The methyltransferase activity of EZH2 makes it an essential epigenetic regulator in the development of embryos, and EZH2 deficiency can lead to the onset of cell differentiation, downregulation of pluripotency-associated stem cell markers, and embryonic growth impairment and lethality.^[31,39]^ Studies have shown that EZH2 plays a significant role in the fate switch of neural stem cells toward the neuronal lineage, regulation of neural stem cell proliferation, and maintenance of stemness. The absence of EZH2 can induce defects in cortical development and learning disabilities.^[40]^ Moreover, aberrant expression of EZH2 participates in the negative regulation of myogenesis by epigenetically silencing muscle-specific markers at the transcriptional level. Induction of muscle cell differentiation can disassociate EZH2 from the promoter region of related genes, accelerating the regeneration and exhilaration of skeletal muscle cells after injury.^[31]^

The AKT/STAT3, Notch, and Wnt signaling pathways are the 3 main pathways EZH2 involved in lipolysis and lipogenesis. A previous study showed that EZH2 can activate STAT3 via methylation, which is mediated by Akt signaling.^[29]^ The phosphorylation of EZH2 induced by the inhibition of Akt signaling significantly reduced STAT3 activity. Aberrant Akt activation is prevalent across cancer lineages, is responsible for cancer cell survival, and is closely correlated with adverse clinical outcomes in patients with cancer, such as glioma, ovarian cancer, lung cancer, and pancreatic cancer. Thus, as a downstream event of Akt signaling, the direct interaction between EZH2 and STAT3 significantly contributes to the oncogenic effect of Akt signaling.^[31,40]^ The Notch signaling pathway has been shown to play an important role in cell lineage determination during development.^[41]^ EZH2 can enhance the activation of Notch signaling by directly upregulating the expression of Notch1 via interaction with the promoter region of Notch1 in a PRC2-independent manner. The Notch-EZH2 mechanism is implicated in the onset, progression, and expansion of the stem cell pool.^[42]^ Several studies have implicated EZH2 in the regulation of the Wnt/β-catenin signaling pathway, which plays a crucial role in cell proliferation, differentiation, and apoptosis.^[43,44]^ Wnt/β-catenin signaling is triggered when a Wnt ligand binds to a 7-pass transmembrane Frizzled receptor and its co-receptor, low-density lipoprotein receptor-related protein 6 or lipoprotein receptor-related protein 5, on the cell membrane. Activation of this pathway induces the accumulation of stabilized β-catenin in the cytoplasm. β-Catenin is then redirected to the nucleus, where it initiates the transcription of downstream target genes. EZH2 can suppress the activation of the Wnt/β-catenin signaling pathway by downregulating the expression of WNT3a and β-Catenin and upregulating the expression of glycogen synthase kinase-3β (GSK-3β), an inhibitor of the Wnt/β-catenin pathway.^[45]^

## 4. Roles of EZH2 in Adipogenesis and Obesity

The most recent study by Wu et al^[46]^ explored the role of EZH2 in the differentiation of white, brown, and beige adipocytes in an EZH2 conditional knockout mouse model and mouse embryonic fibroblasts. The 3 types of adipose tissues are white, brown, and beige. White adipocytes are the most abundant adipocytes in humans and are responsible for the maintenance of metabolic homeostasis by secreting adipokines and lipokines.^[47]^ EZH2F/FPrx1-cre mice have a lower body weight and exhibit a leaner phenotype than wild-type mice. Among the adipose tissues from different regions, the size of adipose tissues predominantly containing white adipocytes is dramatically reduced in EZH2 knockout mice compared to wild-type control mice, indicating that white fat is reduced in the body along with the suppression of EZH2 expression. They then examined the morphological features of white fat and observed that the sizes of the adipocytes and the lipid droplets in the cells were remarkably smaller in EZH2 knockout mice than in control mice. Furthermore, the major markers of adipocyte differentiation, such as PPARγ, fatty acid binding protein 4, and adiponectin, are downregulated in EZH2 knockout mice, which is caused by changes in metabolic factors, including fatty acid β-oxidation gene long chain lipoyl coenzyme A dehydrogenase and carnitine palmitoyl transferase 1b. Therefore, these findings indicate that EZH2 deficiency can induce defects in adipocyte differentiation, at least by promoting the fatty acid β-oxidation.^[46]^ In contrast, EZH2 knockout mice showed better differentiation ability of brown adipocytes and had more beige adipocytes. The EZH2 knockout mice exhibit better tolerance to diet-induced obesity and insulin resistance. In the diet study, the mice were fed a high-fat diet for 8 weeks. The body weight and weight gain rate in knockout mice group were significantly lower than those in the control group. The effect of EZH2 deficiency on insulin resistance was further examined.^[46]^ Consistent with the changes in body weight between the groups, the levels of fasting blood glucose and fasting insulin in EZH2 knockout mice were significantly lower than those in the control group. The blood glucose level in EZH2 knockout mice was remarkably reduced in response to insulin administration compared to that in control mice. Thus, EZH2 can contribute to the development of obesity and insulin resistance when a high-fat diet is consumed.^[46]^ Moreover, an in vitro study in mouse embryonic fibroblasts revealed that EZH2 regulates adipogenesis by exerting enzyme activity of catalyzing DNA methylation. GSK 126 was used as an inhibitor of EZH2 enzyme activity in the induction of adipocyte differentiation in the assay. GSK126 significantly inhibited the formation of lipid droplets and reduced the lipid content in cells. GSK126 notably decreased H3K27me3 and the expression of pro-adipogenic factors without affecting the expression of EZH2. These results demonstrate that positive regulation of white adipocyte differentiation by EZH2 is mediated by H3K27me3 methylation activity.^[46]^

Another recent study by Hng et al^[48]^ established a link between EZH2 and homeodomain-only protein homeobox (HOPX) in regulating the adipogenic fate of MSCs. Quiescent MSCs possess multiple differentiation potentials to differentiate into adipocytes, osteoblasts, chondrocytes, etc.^[49]^ HOPX regulates downstream gene expression through its role as a co-factor that recruits transcription factors to the promoter region of genes.^[50]^ Previous research using global CHIPseq analyses showed that EZH2 represses the expression of HOPX during the differentiation of MSCs into osteoblasts under osteogenic inductive conditions.^[51]^ EZH2 was found to inhibit the expression of HOPX by binding to putative DNA binding sites, preferentially the S3 binding region, in the HOPX promoter in MSCs. They further confirmed that ectopic overexpression of HOPX significantly enhanced the proliferation and inhibited adipogenesis of MSCs.^[48]^ Overexpression of HOPX led to a reduction in the number of mature adipocytes producing lipid droplets and the expression of C/EBPα and adipokines. These results were further confirmed by targeting HOPX with siRNA in the MSCs. Silencing HOPX reversed the inhibitory effect of HOPX on MSCs under adipogenic induction conditions. The suppression of adipogenesis in MSCs by HOPX is mediated by repressing the expression of adipogenesis-associated genes, especially C/EBPα, and co-factors binding to adipogenic suppressors.^[48]^ RNA sequencing results showed that numerous genes were upregulated during the induction of adipogenesis in MSCs, but downregulated in HOPX-enforced overexpression, including 185 genes coding for functional proteins in adipogenesis and 127 genes coding for factors involved in fatty acid metabolism. Because EZH2 is an upstream negative regulator of HOPX, EZH2 can steer the fate of MSCs away from the osteoblastic lineage but toward adipocytes by suppressing the expression of HOPX via epigenetic regulation. In the presence of adipogenic inducers, the expression of HOPX was significantly downregulated, which might be a consequence of synergistic regulation by both adipogenic inducers and EZH2.^[48]^

Recently, Liu et al^[52]^ demonstrated that EZH2 mediates the pro-adipogenic role of circular RNA SAMD4A in obesity. Circular RNAs are a group of small non-coding RNAs implicated in the regulation of gene expression.^[53]^ Emerging evidence shows that circular RNAs are essential for adipogenesis regulation.^[54,55]^ The levels of circSAMD4A were dramatically increased in adipose tissues from obese subjects compared to those in lean visceral adipose tissues from lean controls. It has been shown that the upregulation of circSAMD4A is positively correlated with body mass index in the obese patients and non-remission within one year post bariatric surgery. receiver operating characteristic analysis with a circSAMD4A cut-off ≥2.8 exhibits a high diagnostic performance that is reflected by 93% sensitivity and 88% specificity.^[52]^ Downregulation of circSAMD4A expression mediated by AAV9 significantly reduces fat mass and increases lean mass in obese mouse models. Collectively, circSAMD4A overexpression in adipose tissues correlates with poor prognosis in obese patients, acting as an independent risk factor for predicting clinical outcomes.^[52]^ Further study of the molecular mechanism underlying the pro-adipogenic role of circSAMD4A revealed that circSAMD4A enhances adipocyte differentiation via the miR-138-5p/EZH2 pathway. There was a positive association between the levels of circSAMD4A and EZH2 expressed in the visceral adipose tissues of obese patients. Knockdown of circSAMD4A resulted in a decrease in EZH2 expression, which was rescued by the restoration of circSAMD4A or miR-138-5p inhibition in preadipocytes. When the miR-138-5p mimic was transfected into preadipocytes to overexpress miR-138-5p, the levels of EZH2 were remarkably decreased, which was abrogated by silencing miR-138-5p.^[52]^ Moreover, overexpression of circSAMD4A promoted the luciferase activity of the wild-type EZH2 3’untranslated region, but not the mutated EZH2 3’untranslated region in preadipocytes. However, overexpression of circSAMD4A failed to increase EZH2 expression in cells with miR-138-5p upregulation, whereas miR-138-5p silencing increased EZH2 expression when the expression of circSAMD4A was downregulated. CircSAMD4A was identified as a sponge for miR-138-5p, which downregulates miR-138-5p by direct binding. Thus, CircSAMD4A can induce adipocyte differentiation by stabilizing EZH2 expression by suppressing miR-138-5p.^[52]^

Despite the pro-adipogenic role of EZH2, EZH2 can help MSCs preserve their multipotent differentiation capacity. As previously discussed, β-catenin, stabilized by Wnt signaling, exerts an inhibitory effect on adipocyte differentiation. Sen et al^[56]^ reported that EZH2 is a downstream target of β-catenin that protects the multipotency of MSCs. β-catenin can directly bind to the promoter region of EZH2 to activate the gene transcription of EZH2. They showed that β-catenin prevents both adipogenic and osteoblastic commitment in cytoskeleton-dependent and cytoskeleton-independent manners, respectively. During MSC differentiation, the activation of the Wnt signaling pathway and the adoption of rigidity-dependent characteristics of the cytoskeleton by the cells enhance their commitment to osteoblasts over adipocytes.^[56]^ The inhibition of GSK-3β expression by mechanical strain promotes β-catenin levels in MSCs, which leads to the suppression of adipocyte differentiation. The inhibitory effect of β-catenin on adipocyte differentiation was mediated by inactivation of PPARγ. However, β-catenin also plays an important role in the maintenance of MSCs pluripotency, which is partially mediated by EZH2. During induced osteogenesis in MSCs, inhibition of EZH2 activity by GSK126 significantly promoted the expression of osteogenic genes, which was enhanced by β-catenin knockdown. Inhibition of EZH2 expression or activity results in the loss of β-catenin anti-differentiation capability.^[56]^

## 5. Conclusions and Perspectives

**Figure 3.**
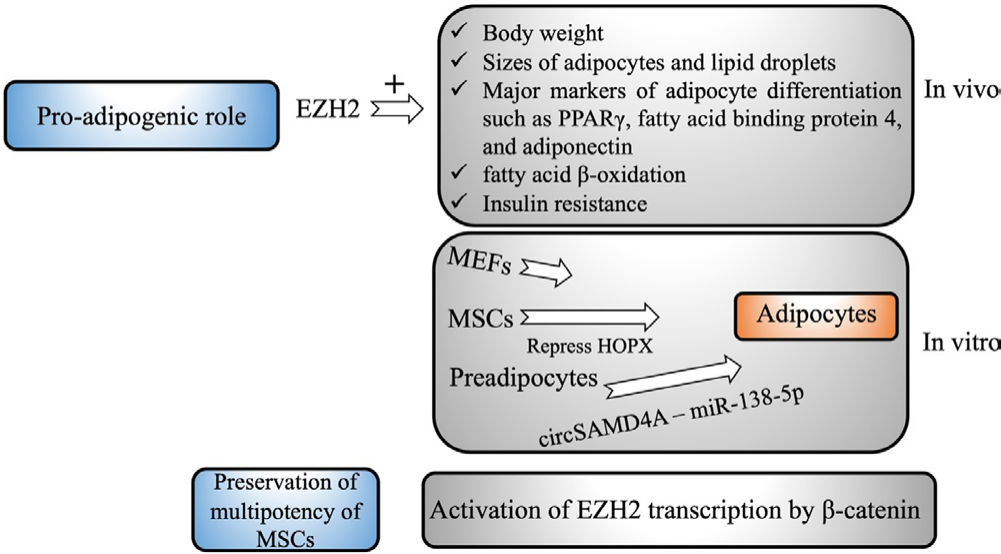
Roles of EZH2 in adipogenesis and obesity. EZH2 = enhancer of zeste homolog 2.

As illustrated in this review, EZH2 has emerged as an important modulator in adipogenesis and obesity. Previous studies have demonstrated that EZH2 expression is implicated in the process of adipocyte differentiation, development of obesity, and preservation of the multipotency of mesenchymal cells (Fig. 3). These data suggest a potential role for EZH2 in obesity and obesity-related metabolic disorders as a diagnostic marker and therapeutic target for the treatment of obesity. During adipogenesis or the progression of obesity, EZH2 acts either as an upstream regulator of adipogenic genes or as a downstream target regulated by adipogenic drivers. However, it is necessary to understand the complexity of the mechanisms underlying the role of EZH2 in the regulatory network of adipogenesis and health conditions related to obesity, such as type II diabetes, heart disease, stroke, high blood pressure, liver disease, gallbladder disease, certain cancers, and sleep apnea.

## Author contributions

**Conceptualization:** Haixia Wang.

Formal analysis: Haixia Wang.

Writing – original draft: Haixia Wang.

Writing – review & editing: Haixia Wang.
